# Appropriate prescribing in nursing homes demonstration project (APDP) study protocol: pragmatic, cluster-randomized trial and mixed methods process evaluation of an Ontario policy-maker initiative to improve appropriate prescribing of antipsychotics

**DOI:** 10.1186/s13012-016-0410-x

**Published:** 2016-03-29

**Authors:** Laura Desveaux, Tara Gomes, Mina Tadrous, Lianne Jeffs, Monica Taljaard, Jess Rogers, Chaim M. Bell, Noah M. Ivers

**Affiliations:** 1 Women’s College Research Institute and Women’s College Hospital Institute for Health Systems Solutions and Virtual Care, Women’s College Hospital, 76 Grenville Ave, Toronto, ON Canada; 2 Li Ka Shing Knowledge Institute, St. Michael’s Hospital, 209 Victoria St, Toronto, ON Canada; 3 Institute for Clinical Evaluative Sciences, 2075 Bayview Avenue, Toronto, ON Canada; 4 Ontario Drug Policy Research Network, St. Michael’s Hospital, 30 Bond St, Toronto, ON Canada; 5 Clinical Epidemiology Program, Ottawa Hospital Research Institute, Ottawa Hospital, Civic Campus, 1053 Carling Ave, Ottawa, ON Canada; 6 School of Epidemiology, Public Health and Preventive Medicine, University of Ottawa, Ottawa, ON Canada; 7 Centre for Effective Practice, 203 College St, Toronto, ON Canada; 8 Department of Medicine, Mount Sinai Hospital and University of Toronto, 600 University Ave, Toronto, ON Canada; 9 Department of Family and Community Medicine, Women’s College Hospital and University of Toronto, 76 Grenville Ave, Toronto, ON Canada

**Keywords:** Academic detailing, Antipsychotic medication, Inappropriate prescribing, Nursing home, Randomized trials

## Abstract

**Background:**

Antipsychotic medications are routinely prescribed in nursing homes to address the behavioral and psychological symptoms of dementia. Unfortunately, inappropriate prescribing of antipsychotic medications is common and associated with increased morbidity, adverse drug events, and hospitalizations. Multifaceted interventions can achieve a 12–20 % reduction in antipsychotic prescribing levels in nursing homes. Effective interventions have featured educational outreach and ongoing performance feedback.

**Methods/Design:**

This pragmatic, cluster-randomized control trial and embedded process evaluation seeks to determine the effect of adding academic detailing to audit and feedback on prescribing of antipsychotic medications in nursing homes, compared with audit and feedback alone. Nursing homes within pre-determined regions of Ontario, Canada, are eligible if they express an interest in the intervention. The academic detailing intervention will be delivered by registered health professionals following an intensive training program including relevant clinical issues and techniques to support health professional behavior change. Physicians in both groups will have the opportunity to access confidential reports summarizing their prescribing patterns for antipsychotics in comparison to the local and provincial average. Participating homes will be allocated to one of the two arms of the study (active/full intervention versus standard audit and feedback) in two waves, with a 2:1 allocation ratio. Homes will be randomized after stratifying for geography, baseline antipsychotic prescription rates, and size, to ensure a balance of characteristics. The primary outcome is antipsychotic dispensing in nursing homes, measured 6 months after allocation; secondary outcomes include clinical outcomes and healthcare utilization.

**Discussion:**

Policy-makers and the public have taken note that antipsychotics are used in nursing homes in Ontario far more than other jurisdictions. Academic detailing can be an effective technique to address challenges in appropriate prescribing in nursing homes, but effect sizes vary widely. This opportunistic, policy-driven evaluation, embedded within a government-initiated demonstration project, was designed to ensure policy-makers receive the best evidence possible regarding whether and how to scale up the intervention.

**Trial registration:**

ClinicalTrials.gov NLM Identifier: NCT02604056.

## Background

Nursing home residents are at a greater risk for inappropriate prescribing as they are more fragile, receive interventions from multiple point-of-care staff, and are unable to detect errors in their medication [1]. Inappropriate prescribing is often linked to polypharmacy and to subsequent adverse outcomes such as increased hospital admissions, increased morbidity, and impaired functionality [2, 3]. In particular, antipsychotic medications (APMs) are known to be associated with cardiovascular events, parkinsonism, falls, and death [4]. Furthermore, APMs are associated with increased morbidity and mortality among elderly residents with dementia [5], signaling the pressing need for solutions to mitigate inappropriate use and encourage appropriate pharmacological and non-pharmacological management of symptoms. In Ontario, Canada, APM prescription rates in nursing homes range from 0 to 67 %, with an average of 33 % of nursing home residents using an APM [6]. Nursing home administration and government officials are facing increasing public and media scrutiny [7] as rates are more than double those in the USA.

Interventions to reduce inappropriate prescribing of APMs among people with dementia in nursing homes have achieved reductions in APM prescribing levels between 12 and 20 % [5]. In a systematic review of interventions to optimize prescribing in nursing homes, academic detailing had the strongest evidence for improvements in prescribing, whereas audit and feedback (AF) interventions had a small to moderate impact on physician practices [1]. For educational interventions to be effective at improving inappropriate prescribing, recommendations suggest they must be implemented through techniques that engage health professionals to consider behavior change, such as academic detailing [1, 8]. AF can act as a complementary strategy by drawing attention to gaps between actual and desired performance, priming the recipients to respond to educational interventions [8]. One prior randomized controlled trial combining quarterly AF plus education around quality improvement demonstrated an overall decrease in resident falls, behavioral symptoms, and pressure ulcers, including among low-risk residents, compared to a control group receiving neither intervention [9].

In Ontario, Canada, policy-makers, including the Ontario Ministry of Health and Long-Term Care (MOHLTC) and Ontario Medical Association (OMA), established an appropriate prescribing working group which is tasked to make recommendations on opportunities to improve prescribing in Ontario. One project that has emerged from this working group is the Appropriate Prescribing Demonstration Project, which involves the evaluation of an academic detailing intervention in nursing homes. Members of the project team are accountable to the policy-makers, and the evaluation approach required policy-maker approval. This partnership and pragmatic evaluation of a policy-maker-driven initiative increases the likelihood that the embedded evaluation will achieve an impact on health service delivery and policy [10]. We designed a pragmatic, cluster-randomized controlled trial that seeks to assess the impact of the intervention with an embedded, mixed methods process evaluation to understand how and why intervention effects were observed and provide insights regarding scale-up.

## Methods/Design

A pragmatic, cluster-randomized controlled trial with an embedded process evaluation of the Appropriate Prescribing Demonstration Project will be undertaken and includes both summative and formative components. The protocol received ethics approval from the University of Toronto and Women’s College Hospital Research Ethics Boards. The trial is registered on ClinicalTrials.gov (NLM Identifier: NCT02604056).

### Setting

In the province of Ontario, many healthcare services are covered under the Ontario Health Insurance Program (OHIP)—a government-run health plan that is publicly funded. OHIP covers medically necessary services provided by physicians, including basic and emergency services, and specialist visits. Within nursing homes, all personal and nursing care is funded by the government, while residents are responsible for accommodation charges such as room and board. Accommodation costs are set by the MOHLTC and are standard across the province of Ontario. Rate reductions are available through a government subsidy for those with low income on a case-by-case basis. The Ontario Drug Benefit Program covers prescription drug costs for individuals who reside in nursing homes, including some nutritional products and diabetic testing agents, provided they are prescribed by an Ontario doctor or other authorized prescriber.

### Trial design

The effectiveness of the intervention will be evaluated using a two arm, pragmatic, cluster-randomized trial, with nursing homes allocated to the full, active intervention (featuring academic detailing offered to each prescriber and team members in the home) or standard quality improvement supports with blinded outcome analysis of administrative data after 6 months. The standard quality improvement supports, which includes individualized AF reports administered by Health Quality Ontario (HQO), represent “usual care” as this initiative has been launched province-wide and therefore a concurrent control arm with no exposure to a quality improvement intervention is not feasible.

### Eligibility

Nursing homes within pre-determined regions of Ontario will be eligible if they expressed an interest in the full intervention. These regions, or hubs, were chosen because they contain a wide variety of nursing home types within a reasonable travel distance (i.e., <100 km). To be eligible for participation, the medical and administrative leads of the nursing homes must agree to and support the project. Exclusion criteria include nursing homes without any prescribers caring for at least 10 residents routinely or nursing homes with fewer than 30 residents.

### Recruitment

A general recruitment email will be distributed by the OMA and MOHLTC to eligible home administrative leads and from other stakeholders (i.e., local physician leads) to medical directors of eligible nursing homes. Identification of additional homes will be done in collaboration with the Local Health Integration Networks and other stakeholders.

The entire nursing home is considered a research participant and so explicit, written consent will be obtained from the medical director and administrative leads from each home. Individual providers and staff members in the home can decide whether and when to engage with the academic detailing intervention.

### Allocation

**Fig. 1:**
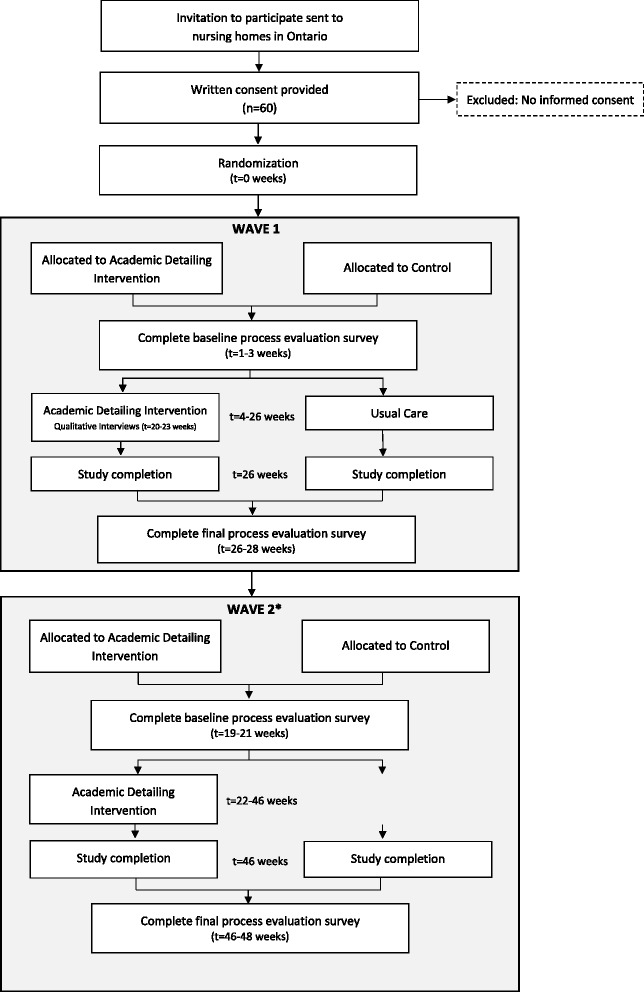
Flow of participants

Participating nursing homes will be allocated to one of the two arms of the study (active/full intervention versus standard AF), with an intended 2:1 allocation ratio. The intervention will be administered in two waves to optimize resource use (i.e., gradual training and phasing-in of program delivery staff) (refer to Fig. 1); therefore, homes will be allocated to the intervention in two waves. Nursing homes that share prescribers will be grouped together and allocated as a unit to avoid contamination. Homes (or groups of homes) will be randomized in two waves after stratifying for geography, as well as baseline APM prescription rates and size (categorized as above or below the median), to promote a balance of characteristics between the two arms. A 2:1 allocation ratio will be used to promote recruitment and to satisfy the requirements of the trial sponsor without drastically affecting sample size requirements. Allocation will be conducted by an independent statistician blinded to home identity using computer-generated random numbers.

### Interventions

#### Standard quality improvement supports/usual care

Policy-makers previously determined that all nursing home physicians in the province would be provided with the opportunity to access online practice reports (serving as AF) that would confidentially describe their prescribing practices for APMs, compared to regional and provincial data representing their (de-identified) peers. Starting in autumn 2015, in concert with the initiative outlined in this proposal, HQO will be producing confidential practice reports using data from the Institute for Clinical Evaluative Sciences (ICES), a not-for-profit research institute with a secure and accessible array of Ontario’s health-related data, to describe selected quality indicators for physicians working in nursing homes. This will initially focus on appropriate prescribing of APM for older men and women and provides data on how their APM use compares to others in the province. The report also includes additional information regarding the clinical and demographic features of their nursing home resident roster. Receipt of the reports is voluntary. To receive the reports, physicians must sign up and confirm their identity. They will then receive updated reports quarterly, which they can download from HQO’s secure website. In addition, HQO will be launching voluntary, virtual “communities of practice” to enable sharing of best practices across homes and providers throughout the province.

#### Academic detailing (active intervention)

The academic detailing intervention will be delivered by registered health professionals (e.g., nurses or pharmacists) who have received intensive training to support providers in nursing homes, to address challenges and opportunities to improving prescribing practices and overall quality of care. Academic detailing is a method of service-oriented educational outreach for front-line clinicians which combines the interactive, one-on-one communication approach of industry detailers with the evidence-based, non-commercial information of academia [11]. The intervention requires the Academic Detailers to have direct and ongoing contact with the nursing homes including administrators, providers, and staff from the time of launch through to program completion. Given the diverse target audiences that will likely be engaged within the homes, the Academic Detailer will work to understand the context, barriers, capacity, and needs of each home to ensure the service being provided by the detailer is relevant, meaningful, and appropriate. The intervention then includes the provision of the following different “types” of visits made by the Academic Detailer: meetings (likely with administrators or home leadership to describe the program and value), presentation (large group, inter-professional audience), group visit (typically 2–6 providers), and one-on-one visits (“traditional” academic detailing visits). In addition, the Academic Detailer provides important service to the nursing homes and providers between visits (i.e., by responding to questions via email or phone) to respond to the needs and build trust/credibility over time. To optimize resource allocation, the academic detailing intervention will be administered in two waves (October 2015 and February 2016), each lasting a duration of 6 months.

The Centre for Effective Practice, a Toronto-based federal non-profit organization retained by the OMA/MOHLTC based on their experience with academic detailing, will manage the academic detailing intervention. Best practices in training of the Academic Detailers and in the execution of the program will be applied (refer to Appendix). Given the nature of the program as a demonstration project for the purposes of understanding feasibility in the Ontario context, it is critical to ensure a pragmatic approach to the delivery of the intervention. Therefore, the number of visits per provider or home per topic and the length of visits will not be pre-defined or standardized. Intervention fidelity and acceptability metrics will be evaluated through the embedded process evaluation.

### Data collection

Outcomes will be assessed using population-level administrative databases linked through unique, encrypted patient identifiers at the ICES. Data will be compiled from the following databases: (1) the Ontario Drug Benefits (ODB) database, covering all prescription medications dispensed to residents in nursing homes; (2) the Canadian Institute for Health Information (CIHI) databases covering all inpatient hospitalizations and emergency department visits; (3) the Ontario Health Insurance Plan (OHIP) database, covering physician billings for procedures and consultations; (4) the Registered Persons Database covering demographic information including date of death; and (5) the Continuing Care Reporting System (CCRS) database for clinical and demographic information on nursing homes residents collected using the Resident Assessment Instrument (RAI). Each nursing home resident in the province receives a “full” RAI assessment within 2 weeks from admission date, every 12 months from previous full assessment or any other time when the clinical condition of a resident changed considerably. Each resident also receives a shorter “quarterly” assessment every 3 months. For each resident, the CCRS database holds demographic and clinical information, including data regarding clinical diagnoses (e.g., dementia, psychosis), dispensing of APMs, and occurrences of falls. In addition, a number of validated outcome scales can be derived from the CCRS data, including those related to activities of daily living (ADLs), aggressive behaviour, pain, and mood.

### Outcomes

The primary prescribing outcome is the number of days the resident was provided an APM in the previous week, as determined from the CCRS database. Secondary prescribing outcomes include mean APM dose (over the previous month) and prevalence of antipsychotic, benzodiazepine, antidepressant, and acetaminophen prescribing (defined as any prescription during the previous month), as determined from the ODB database. All primary and secondary prescribing outcomes will be assessed at baseline (pre-intervention) and at 3 and 6 months post intervention. Baseline is defined as the week or month immediately prior to randomization, while post-intervention measurements are defined as the third and sixth months after randomization. These outcomes will be assessed on all residents who are alive at the time of the assessment and who were present in the home for the full duration of the prior week/month (i.e., repeated cross-sectional within-cluster data).

Secondary clinical outcomes will be extracted from the RAI, including ADLs (measured by the ADL long-form scale) [12]; pain (pain scale) [13]; depression (depression rating scale) [14]; number of falls in the past month; and the extent of aggressive behaviour [15]. These will be assessed at baseline and at 3 and 6 months post intervention. The most current data collected immediately prior to the implementation of the intervention, and at each post-randomization time point, will be used, regardless of mortality status at the subsequent time point.

Health care utilization outcomes will be assessed at baseline and at 6 months post intervention and include the number of ER visits and hospitalizations during the previous 6 months. A longer duration is required to allow adequate time for the intervention to have an effect on these outcomes. All residents present in the homes at any time during the study will contribute to the analysis using person-time of follow-up.

### Data analysis

Outcome evaluations will use de-identified data available from the ICES and will be conducted by analysts blinded to group allocation. All evaluations will be reported at the aggregate level, and the confidentiality of nursing homes, residents, and providers will be protected.

#### Impact of the intervention on clinical outcomes

Descriptive statistics will be calculated for all variables of interest: continuous variables with a normal distribution will be described using means and standard deviations (medians and inter-quartile ranges will be used in the case of skewed distributions), whereas categorical variables will be summarized using frequencies and proportions. All analyses will be conducted under the principles of intention-to-treat analysis and will be conducted using SAS v.9.3. Statistical significance will be assessed at the 5 % level.

The primary outcome (days dispensed APM in the previous week) measured at baseline, 3 months, and 6 months will be analyzed using generalized linear mixed effects regression with multinomial distribution and cumulative logit link. The fixed effects of time, intervention, and intervention by time will be the main variables of interest. To account for the staggered implementation of the intervention, wave will be included as a fixed effect. Random intercepts and slopes will be specified to account for intracluster and inter-period correlations. To maximize statistical efficiency, the analysis of prescribing outcomes will adjust for the stratification factors (hub, baseline APM prescription rates, and size) as well as public/private home ownership and the following resident covariates: age, sex, levels of function, and aggressive behaviour (using multiple imputation for missing covariates if necessary). The proportional odds assumption will be evaluated using the score test. The effect of the intervention at 6 months will be expressed as cumulative odds ratio (OR) with 95 % confidence interval (CI), which expresses the odds of having fewer days with APM dispensed in the intervention arm compared to the control arm.

The dichotomous prescribing outcomes (i.e., any APM prescription in the last month) at baseline, 3 months, and 6 months will be analyzed using generalized linear mixed effects regression with binomial distribution and logit link function. Fixed and random effects will be specified as described for the primary outcome. The fixed effects of time, intervention, and intervention by time will be the main variables of interest. Dose equivalent of APM dispensed in the past month at baseline, 3 months, and 6 months will be analyzed using linear mixed effects regression with normal distribution and identity link. Fixed and random effects will be specified as described for the primary outcome. The effect of the intervention will be described as difference in change from baseline to 6 months using adjusted least square means with 95 % CI.

Secondary clinical outcomes measured as continuous variables (e.g., ADL, depression score) will be analyzed using linear mixed effects regression with normal distribution and identity link. Fixed and random effects will be specified as described for the prescribing outcomes. The effect of the intervention will be expressed as adjusted least square mean differences with 95 % CI. Secondary clinical outcomes measured on an ordinal scale (e.g., pain scale) at baseline, 3 months, and 6 months will be analyzed using generalized mixed effects regression as described for the primary outcome but using multinomial distribution and cumulative logit link. Presence of any falls in the past 30 days will be analyzed at baseline, 3 months, and 6 months using generalized linear mixed effects regression with binomial distribution and logit link, as described for the primary outcome. In the intention-to-treat analyses for the secondary clinical outcomes (e.g., ADL, falls, pain), the potential effect on inferences of residents who died will be examined using sensitivity analyses under conservative assumptions: for example, residents who died during the interval will be assigned the worst possible score. Depending on the extent of resident turnover within homes, additional analyses for primary and secondary outcomes will explore inclusion of subject-specific random effects to account for repeated measures on the same resident over time (i.e., a cohort rather than cross-sectional design).

Health care utilization outcomes will be analyzed at 6 months using generalized linear mixed effects regression with Poisson or negative binomial distribution and log-link, with log person-time of follow-up as an offset term. The following fixed effects will be included: intervention, phase, and stratification variables, as well as the rate of health care utilization at the home 6 months prior to the intervention. Resident covariates will be adjusted for as specified for the primary outcome. Home will be included as a random effect.

The effect of the intervention will be expressed as adjusted rate ratio (RR) with 95 % CI. Potential effect modification by home ownership status, size of the home (<65 beds, 65–129 beds, or >129 beds), location (urban/rural), specialist utilization (proportion of residents with geriatrician or psychiatrist consultation within 6 months), plus primary physician characteristics (sex, years of experience), as well as resident characteristics (age, sex, history of psychosis, diagnosis of dementia, time at facility, levels of function, and aggressive behaviour, plus whether a geriatrician or psychiatrist provided a consult) will be explored by including interactions between these variables and treatment. A planned sensitivity analysis will restrict the academic detailing cohort to physicians who also accessed the AF to examine for synergy (i.e., interaction) in the full intervention group.

#### Economic evaluation

We will work with the program delivery teams to determine the costs for start-up and implementation. Average costs will be presented as $/nursing home, $/nursing home physician, and $/nursing home resident. A cost-benefit analysis will compare costs of the intervention with direct savings related to changes in prescribing (if any) as well as the cost per type of clinical outcome achieved (e.g., $/Rx change). A more detailed economic evaluation modelling overall health care costs was considered out of scope.

### Power calculation

Our target sample size of 60 nursing homes was determined primarily by pragmatic considerations and logistical constraints. Specifically, the contract between the MOHLTC and the Centre for Effective Practice specified that 40 nursing homes would receive academic detailing.

After grouping homes sharing physicians together, we anticipate a total of 45 independent clusters. For our primary outcome measured as an ordinal variable (number of days with APM prescription in the past 7 days), 45 clusters with 2:1 allocation will yield 80 % power to detect a minimally important difference (cumulative odds ratio) of 0.6, assuming an intracluster correlation coefficient (ICC) of 0.01 and an average of 120 residents per home. If we found that 30 % of control arm residents have 7 days of APM prescriptions, a cumulative odds ratio of 0.6 would correspond to an absolute reduction of 10 % in this proportion.

To ensure adequate power to detect a clinically important difference in our secondary prescribing outcome, we used a simulation study with 1000 simulation runs to determine the required effect size. Assuming a conservative ICC of 0.1, an average of 120 residents per home, and a control arm proportion of 30 %, we will have 83.4 % power to detect an OR of approximately 0.75 for our secondary prescribing outcome at 6 months (corresponding to a reduction in the intervention arm to approximately 25 %).

### Embedded process evaluation

The process evaluation involves a mixed methods approach using quantitative prescriber surveys, document analysis, and individual qualitative interviews. To understand the intervention’s mechanism of change, we will survey participating clinicians and nursing home administrators in both groups prior to intervention delivery and again 6 months later to measure theoretical constructs targeted by our intervention and changes in these constructs associated with the intervention [16]. We will assess constructs based on the COM-B model (capability, opportunity, and motivation) [17], which are specifically targeted by the intervention. These constructs will be assessed for each of the targeted clinical behaviours. Questions will include validated items from the Determinants of Implementation Behaviour Questionnaire [18] as well as measurements of self-efficacy and coping planning, two factors which have been shown to influence clinician behaviour [19].

To evaluate how and why the intervention achieved the effects observed, we will employ an embedded single case design with cross case synthesis to gain an in-depth understanding of intervention fidelity, the mechanisms of action, and the conditions and factors associated with implementation of the intervention. The embedded design enables the examination of interactive processes and contextual features through various units of analysis derived from a structured approach to develop a program theory [20–22]. The Consolidated Framework for Implementation Research (CFIR) [23] and Normalization Process Theory (NPT) [24] will be used to guide and structure data collection methods and data analysis. The CFIR provides a comprehensive taxonomy of defined constructs that are likely to influence implementation [23]. Its application facilitates the identification of active components and further explores the setting in which the intervention is delivered. NPT is an established framework for understanding how and whether complex interventions become embedded in health care practice—that is, normalized [24]. The inclusion of both frameworks provides a complementary approach as the application of CFIR helps to capture contextual factors that influence implementation and effectiveness while NPT facilitates the understanding of the mechanisms that underlie sustained impact. This approach is ideally suited to this study as it entails numerous individuals, professionals, unit, and organizational and health system features that may impact the effectiveness of the AF + AD intervention in selected nursing homes.

### Recruitment

A quantitative survey will be administered by the research team to the nursing home leadership across all participating sites. At the time of study launch and after 6 months, the research team will distribute the survey, asking home leadership to complete it themselves in addition to forwarding the survey to the prescribers and pharmacists. A series of weekly reminders to home leadership will be sent using a modified Dillman approach [25], for a total of 3 weeks, to encourage completion.

A sub-sample of 4–5 nursing homes from the intervention group (AF + AD) will be selected for the qualitative process evaluation. These homes will be selected to maximize sampling variation, including prescribing rates, size, ownership, and resident characteristics. Initial contact will be from the investigative team with each home’s Senior Management and/or Director of Care/Nursing. A member of the research team will inform participants of the nature of the study, which is to understand how stakeholders respond to and interact with the interventions, to explore any unintended consequences, and to examine experiences associated with the implementation of the academic detailing intervention. As a first wave of recruitment for health professionals in the embedded process evaluation, the leadership team of each home will be asked to distribute a letter of information via email about this aspect of the larger study to the prescribing health professionals in the 4–5 facilities where the case studies will be focused. The email message will have pertinent information of the nature of the study. As a second phase of recruitment, if response is less than 25 %, a member of the research team will reach out to home administrators by telephone. The third and final phase of recruitment will consist of purposive sampling of prescribers and pharmacists associated with the home, using snowball techniques to seek providers with various perspectives on APMs. This method would entail existing participants to identify potential participants among their known acquaintances. All academic detailers will be invited to participate in an interview.

### Data collection

To understand the intervention’s mechanisms of change, we will survey participating clinicians and nursing home administrators in both groups prior to intervention delivery and again following the completion of the intervention to measure theoretical constructs targeted by our intervention and changes in these constructs associated with the intervention [16]. We will assess constructs based on the COM-B model (capability, opportunity, and motivation) [17], which are specifically targeted by the intervention. These constructs will be assessed for each of the targeted clinical behaviours. Questions will include validated items from the Determinants of Implementation Behaviour Questionnaire [18] as well as measurements of self-efficacy and coping planning, two factors which have been shown to influence clinician behaviour [19].

We will also interview home administrators and prescribers, including physicians, nurse practitioners, and consultant pharmacists. Interviews will be used to understand how participants respond to and interact with the interventions and also to explore their experiences associated with the implementation of the academic detailing intervention, including any unintended consequences. Academic Detailers will also be invited to participate in an interview in order to explore their experiences associated with the implementation of the academic detailing intervention. The interviews will be guided by the detailed program theory (which itself will be refined iteratively), the CFIR [23], and NPT [24]. The interview guides will be pilot tested prior to their full-scale use. It is expected interviews will last between 30 and 90 min. Interviews will be digitally audio taped and transcribed verbatim by an external third party.

### Data analysis

Analysis of baseline survey data will include descriptive statistics and exploration of the association between process variables and receiving the intervention as a preliminary test of the program theory. This will be conducted by a statistician without knowledge of group allocation. Measures of capability, opportunity, and motivation will be calculated as the mean of the measure item scores. We will use analytical methods previously developed to test for differences between groups on hypothesized targeted constructs, controlling for baseline differences [16]. Mediation models will be used to test whether intervention effects on behaviour are mediated through the targeted theoretical constructs.

Training materials will be analyzed using Charmaz’s textual analysis method [26] and compared to interview findings to evaluate intervention fidelity. Interviews will be analyzed using the framework method [27, 28], with CFIR constructs applied as pre-defined deductive codes. Open coding will be applied as required by the data to allow for the emergence of themes not captured across CFIR constructs. Qualitative findings will be considered through the lens of NPT in order to identify potential mechanisms by which the intervention’s observed effect becomes part of routine practice. In the final analytical phase, the research team will prepare a comprehensive case study database with the datasets from each of the research questions [29]. We will employ a variety of strategies to ensure fidelity and credibility of the data: (1) using multiple sources of data, writing a chain of evidence that describes all elements of the case study database and a description of the derivation of the evidence from initial research questions to ultimate case study conclusions, and having key informants review the case study draft by having collaborators participate in the triangulation analysis and the return of findings (construct and external validity); (2) examining points of convergence (pattern matching) and divergence (examining alternative explanations) within and among the various datasets (internal validity through cross comparative analyses); and (3) creating a case study database with a chain of evidence and having a stepped analysis process whereby there is an initial independent review of the data by at least three reviewers who then meet to reach consensus around the common themes (reliability) [30]. The framework approach ensures a systematic approach to summarizing and classifying the data, facilitating a comprehensive review of participant narratives while encouraging a higher-level conceptual analysis [28]. Interconnected analytic stages allow for the constant refinement of themes while moving toward the development of an overarching conceptual framework of how the intervention works and why.

### Trial status

The first wave of detailing has been completed. The second wave of intervention delivery commenced in March 2016. Cases for the qualitative aspect of the process evaluation were identified in February 2016, with interviews occurring through February and March 2016.

## Discussion

Ontario has one of the highest rates of APM prescriptions in Canada, with approximately 30.5 % of nursing home residents receiving inappropriately prescribed APMs in the absence of diagnosed psychosis [31]. Nursing home residents may receive up to four times as many prescriptions compared to the elderly in the community [1], increasing the risk of drug-to-drug interactions and adverse outcomes [32, 33]. Prior research has indicated that inappropriate decision-making, limited knowledge of APM risks and benefits, limited personnel, excess employment of temporary nurses, and the employment of nurses with weak interpersonal skills contribute to an increase in the perceived need for psychotropic drug use [34]. To address suboptimal prescribing practices, interventions must consider these factors and support prescribers to implement alternative approaches [8, 35].

In nursing homes and other settings, academic detailing is an effective intervention to improve inappropriate prescribing [1, 8]. However, the magnitude of effects observed in studies varies widely and a local program evaluation is needed to determine whether and how to scale up the intervention. The results of the embedded process evaluation will help identify the core components and the contextual factors that influence its success, providing an overarching understanding of how the intervention works, for whom, and in what circumstances. By exploring both causal attribution and causal explanation, this program evaluation will have significant implications for healthcare administrators, government agencies, and other stakeholders seeking to use academic detailing, alone or combined with other quality improvement strategies, to support large-scale initiatives to scale up and support quality improvement.

### Ethics approval and consent to participate

Ethics approval has been obtained from the University of Toronto Research Ethics Board and the Women’s College Hospital Research Ethics Board.

## Appendix

Academic detailing best practices.

## Abbreviations

AD: academic detailing
ADL: activities of daily living
AF: audit and feedback
APM: antipsychotic medication
CCRS: Continuing Care Reporting System
CIHI: Canadian Institute for Health Information
CFIR: Consolidated Framework for Implementation Research
CI: confidence interval
ER: emergency room
HQO: Health Quality Ontario
ICC: intracluster correlation coefficient
ICES: Institute for Clinical Evaluative Sciences
MOHLTC: Ontario Ministry of Health and Long-Term Care
NPT: normalization process theory
ODB: Ontario Drug Benefits database
OHIP: Ontario Health Insurance Program
OMA: Ontario Medical Association
OR: odds ratio
RAI: Resident Assessment Instrument
RR: rate ratio
